# Simultaneous Quantification of 25 Fentanyl Derivatives and Metabolites in Oral Fluid by Means of Microextraction on Packed Sorbent and LC–HRMS/MS Analysis

**DOI:** 10.3390/molecules26195870

**Published:** 2021-09-28

**Authors:** Flaminia Vincenti, Camilla Montesano, Svetlana Pirau, Adolfo Gregori, Fabiana Di Rosa, Roberta Curini, Manuel Sergi

**Affiliations:** 1 Department of Chemistry, Sapienza University of Rome, 00185 Rome, Italy; flaminia.vincenti@uniroma1.it (F.V.); svetlanapirau@hotmail.com (S.P.); roberta.curini@uniroma1.it (R.C.); 2 Department of Public Health and Infectious Disease, Sapienza University of Rome, 00185 Rome, Italy; 3 Department of Scientific Investigation (RIS), Carabinieri, 00191 Rome, Italy; adolfo.gregori@carabinieri.it (A.G.); fabiana.dirosa@carabinieri.it (F.D.R.); 4 Faculty of Bioscience and Technology for Food, Agriculture and Environment, University of Teramo, 64100 Teramo, Italy

**Keywords:** fentanyl, oral fluid, microextraction on packed sorbent, LC–HRMS/MS

## Abstract

Fentanyl and fentalogs’ intake as drugs of abuse is experiencing a great increase in recent years. For this reason, there are more and more cases in which it is important to recognize and quantify these molecules and related metabolites in biological matrices. Oral fluid (OF) is often used to find out if a subject has recently used a psychoactive substance and if, therefore, the person is still under the effect of psychotropics. Given its difficulty in handling, good sample preparation and the development of instrumental methods for analysis are essential. In this work, an analytical method is proposed for the simultaneous determination of 25 analytes, including fentanyl, several derivatives and metabolites. OF was collected by means of passive drool; sample pretreatment was developed in order to be fast, simple and possibly semi-automated by exploiting microextraction on packed sorbent (MEPS). The analysis was performed by means of LC–HRMS/MS obtaining good identification and quantification of all the analytes in less than 10 min. The proposed method was fully validated according to the Scientific Working Group for Forensic Toxicology (SWGTOX) international guidelines. Good results were obtained in terms of recoveries, matrix effect and sensitivity, showing that this method could represent a useful tool in forensic toxicology. The presented method was successfully applied to the analysis of proficiency test samples.

## 1. Introduction

Fentanyl and its analogues are increasingly being used as drugs of abuse worldwide. In the US, an impressive wave of opioid overdose cases is currently being driven by these molecules [1], which may be classified as synthetic opioids. Fentanyls have also been implicated in deaths in Europe and especially in northern countries (Estonia, Latvia and Sweden) [2]. As a consequence, fentanyl detection has increased not only in seized drugs and postmortem specimens but also on the roadside [3,4]. Increasing reports of the use of fentanyls in driving under the influence of drugs (DUID) cases can be viewed as a major public safety issue that requires the development of suitable analytical methods for roadside testing.

Oral Fluid (OF) is an alternative matrix in analytical toxicology, especially suitable for DUID testing and for workplace applications [5]. As a matter of fact, OF is an exceptional matrix due to its simple and noninvasive sample collection and good correlation with blood concentration, allowing the detection of recent drug intake [6]. On the other hand, OF is a rather complex matrix, protein content is lower when compared to blood, but viscosity, interindividual heterogeneity and possible presence of food debris, etc., make it a difficult sample to deal with [7]. Sample preparation is then an essential step; traditional techniques such as solid phase extraction (SPE) and liquid–liquid extraction (LLE) are still the most diffused for OF pretreatment prior to liquid chromatography–mass spectrometry (LC–MS) analysis but miniaturized techniques are gaining popularity. The increased interest for these alternative preparation techniques arises from a number of advantages, including short extraction time, potential automation, minimum reagent and sample consumption; this last aspect is of particular relevance for OF analysis due to the small amount of samples typically sampled [8]. For what concerns the determination of fentanyl and/or its analogues in this matrix, a few methods have already been published. These include two recent publications from Palmquist et al. [3,9] and a number of papers comprising fentanyls in combination with other opioids [10] and new psychoactive substances (NPS) [11,12,13]. In all of these published methods, traditional sample preparation techniques such as dilution, SPE or LLE were used.

In the present paper, a new method for the determination of fentanyl and 24 among the most diffused analogues in the drugs market, was developed. Sample preparation was based on microextraction by packed sorbent (MEPS), a miniaturized SPE technique which is gaining increasing attention. In MEPS, the sorbent bed is reduced to a few milligrams and inserted in a syringe barrel (100–250 μL), the technique features a short extraction time, reduced sample and solvent consumption and can be automatized. The suitability of MEPS for OF illicit drug testing was previously demonstrated for a number of substances [14,15,16,17] but, to the best of our knowledge, this is the first application to fentanyls. The procedure has been fully validated according to the Scientific Working Group for Forensic Toxicology (SWGTOX) guidelines [18]. For proof of applicability, the method was applied to authentic oral fluid samples received as part of an interlaboratory proficiency test.

## 2. Results and Discussion

### 2.1. HPLC–HRMS/MS

To obtain a complete fragmentation pattern of the analytes, different runs were performed in full-scan data-dependent acquisition mode, each one with three different collision energies (CE) as described in a previously published method [19].

With regard to liquid chromatographic separation, it was decided to work with a mixed sorbent column on the basis of previous experiences with this family of compounds [20]. The mobile phases were selected thanks to the expertise gained in the field of NPS analysis and opioids; in particular, phase A, constituted by 10 mM ammonium formate acidified with 0.1% of HCOOH, was evaluated to perform a better ionization of all target analytes in the H-ESI source, as previously described by Montesano et al. [20], while phase B was chosen since in the literature it is widely demonstrated that mixing different percentages of MeOH and MeCN can improve the chromatographic separation of several types of analytes if compared with the separation provided by the use of single solvents [21]. Different ratios of the two solvents were tested and the necessary peak resolution was obtained by using MeCN–MeOH with a ratio 50:50 (*v*/*v*).

**Figure 1:**
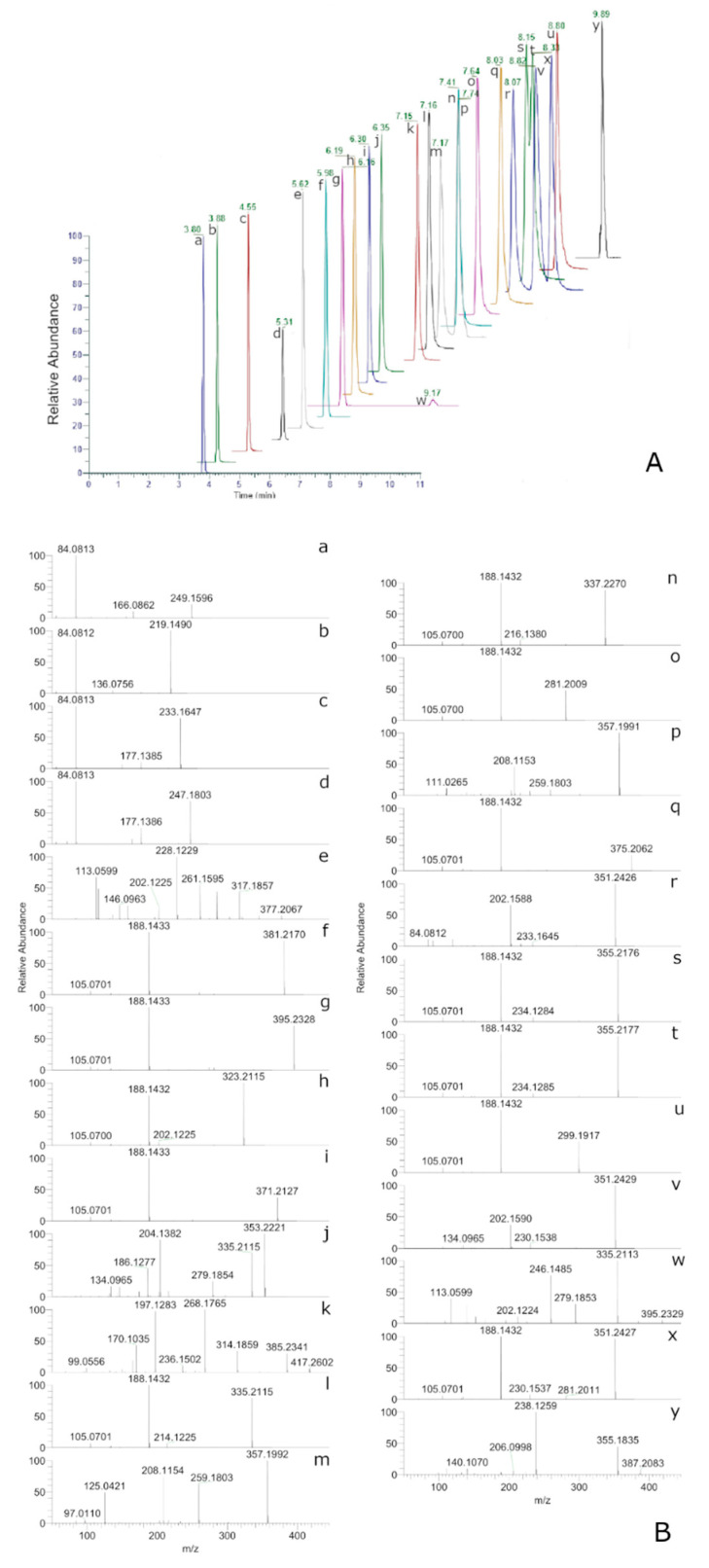
Chromatogram containing the extracted ion currents (**A**) and fragmentation spectra of target analytes, Methoxyacetyl Norfentanyl (a), Acetylnorfentanyl (b), Norfentanyl (c), +/−trans-3-methylnorfentanyl (d), Remifentanyl (e), Butyrylfentanyl Carboxy Metabolite (f), Valerylfentanyl Carboxy Metabolite (g), Acetylfentanyl (h), Ocfentanyl (i), Beta-hydroxyfentanyl (j), Alfentanyl (k), Acrylfentanyl (l), alfa-methylthiofentanyl (m), Fentanyl (n), 4-ANPP (o), +/−cis-3-methylthiofentanyl (p), Furanylfentanyl (q), +/−cis-3-methylfentanyl (r), para-Fluorofentanyl (s), ortho-Fluorofentanyl (t), Despropionyl para-Fluorofentanyl (u), alfa-methylfentanyl (v), Carfentanyl (w), Butyrylfentanyl (x) and Sufentanyl (y) (**B**).

The combination of the mixed column and the described mobile phases led to good performances both in term of separation and run time, allowing complete elution of analytes in 9.9 min. As a matter of fact, using a mixed C18-PFP column it was possible to chromatographically separate some isomers, such as methoxy acetyl fentanyl and beta-hydroxy fentanyl (m/z = 353.2220), and also o- and p-fluoro fentanyl or trans and cis-3-methyl norfentanyl, which possess the same precursor and fragments accurate mass. In the two latter cases, since the fragmentation pattern at the selected CE was exactly the same, to identify the right isomer, standard solutions of the single analytes at 5 ng mL^−1^ were injected in the LC–HRMS/MS system. A chromatogram containing the XICs of all target analytes is reported in Figure 1.

### 2.2. Sample Collection and Sample Preparation

Sample collection was performed by means of passive drool to avoid the use of any stimulant or swab and to better reflect drug concentrations in excreted saliva since expectoration increases the rate of salivary excretion to a minor extent [5]. In order to explore the variability between different subjects, samples were taken from six volunteers of both sex, who followed different and heterogeneous diets.

**Figure 2:**
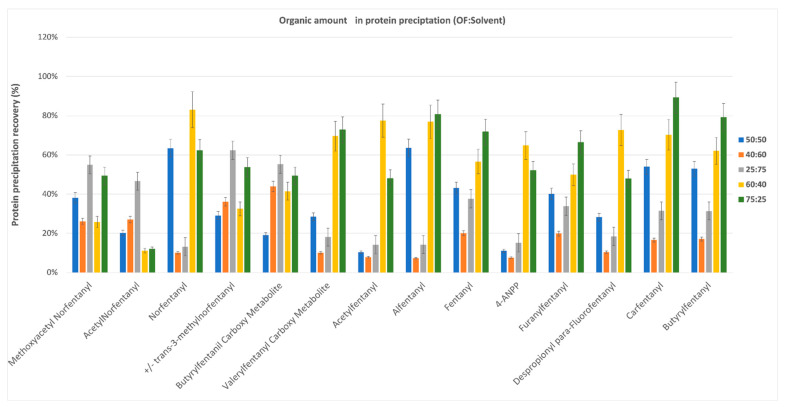
Different ratio between OF and solvent tested for the protein precipitation, i.e., 50/50, 40/60, 25/75, 60/40, 75/25 (OF/solvent).

An initial protein precipitation was performed on each sample to avoid the risk of plugging the BIN. Different types of solvents or mixtures, in different proportions and volumes, were tested to reach the best compromise between effective precipitation and non-excessive dilution of the initial sample. To this aim, methanol, acetonitrile and a mixture of methanol/acetonitrile 50/50 (*v*/*v*) were evaluated. Different ratios between OF and solvent were tested, i.e., 50/50, 40/60, 25/75, 60/40, 75/25 (OF/solvent). Similar good results were obtained with the ratios 60/40 and 75/25 as shown in Figure 2. The ratio 75/25 was chosen considering the dilution factor and the performances obtained.

Developing environmentally sustainable sample preparation techniques, with reduced use of toxic organic solvents is crucial and, in this context, microextraction techniques are gaining increasing interest; in addition, for alternative matrices such as OF, the available amount of a sample is generally low so that downscaling traditional extraction techniques is particularly useful. In fact, microextraction makes it possible to minimize the use of organic solvents, but also to significantly reduce the volume of sample required, analysis time and operating costs. For all these considerations, MEPS was considered a suitable technique for OF extraction with both classic drugs and NPS [16,17]. The same needle can be used for hundreds of samples, so an effective washing must be considered. This technique, similarly to classic SPE, involves different steps, i.e., conditioning of the sorbent material, loading of the sample, washing and finally the elution step to elute the target analytes and eventually enrich the sample. In this work, the sorbent used was a silica-based sorbent material, specifically C18, which was chosen for the great affinity of fentanyl and its derivatives for this solid phase. With regards to sample loading, it was important to define the maximum amount of organic solvent that did not negatively affect retention of the target analytes. This parameter was evaluated through the analysis of the residual solution after loading; the ratio of aqueous/organic solvents tested were 70/30, 80/20 and 90/10. The ratio 90/10 provided the best result, in fact the unloaded amount was between 1% and 4%. For this reason, considering that the amount of organic solvent used for preliminary protein precipitation was greater than 10%, the sample was diluted to reach this ratio before performing the extraction step. In order to reduce the interpersonal variability in terms of OF pH, a buffer was added at this stage. The results were promising and, also, taking into account that the pH of the solution has a great influence on analytes retention on the solid phase, the addition of a buffer solution was crucial to increase analyte recoveries. Thus, 100 μL of a buffer solution, pH 11 were added to 100 μL of the supernatant obtained following the protein precipitation step. pH value was chosen considering the analytes protonation status to increase the compatibility with the MEPS sorbent.

Once the most suitable condition for sample loading had been defined, the washing step was evaluated. From the tests carried out during the sample loading phase, it was clear that to avoid analytes loss, the organic solvent used in the washing step could not be greater than 20%. Experiments were conducted with both MeCN and MeOH. Similar results were obtained for both organic solvents, while the best compromise between the loss of the target analytes and an effective elimination of interfering compounds, was 80/20 H_2_O/MeOH, and it was therefore selected as the washing solution. As concerns the elution, in order to enrich the sample, the volume of the solvent was set to 50 μL. MeOH acidified with different amounts of HCOOH was tested. An increase in the percentage of HCOOH led to an increase in extraction up to the maximum limit corresponding to 1%, so MeOH 1% HCOOH was chosen as the MEPS extraction solvent. To avoid the chromatographic peak fronting, the samples were subsequently diluted 1/1 with water before injection into the HPLC system. 

### 2.3. Validation

Following the criteria reported by the Commission decision 2002/657/EC concerning targeted MS/MS analysis, two characteristic fragment ions were selected for each analyte. For each analyte, the relative intensities of product ions, expressed as their ratio, were calculated, and it was verified that the ratio obtained from the analysis of the matrix sample was the same as that calculated from the analysis of a standard solution at the same concentration. For analyte identification, the accurate mass of the precursor ion and of both fragment ions at the same retention time was taken into account, with a maximum tolerance of 0.1%, as suggested by the European guidelines for workplace drug testing in oral fluid [22].

LODs and LOQs were experimentally calculated by analyzing blank OF spiked with the standard solution at decreasing concentrations. LODs were between 0.05 and 0.50 ng mL^−1^ while LOQs were between 0.10 and 1.0 ng mL^−1^. The values for each analyte are reported in Table 1. The obtained values are lower than those previously reported in the literature [3,9]. In the literature, the absence of cut-off values for NPS is widely discussed so that it is common to refer to real case concentrations to evaluate a suitable range of linearity for a new method [23]. Palmquist et al. observed, in OF from probationers/parolees, fentanyl concentrations ranging from 1.0 to 104.5 ng mL^−1^ and, as expected, lower concentrations for the metabolite 4-ANPP that was found in a range from 1.2 to 5.7 ng mL^−1^. Calibration curves linearity was calculated in OF and was suitable for all of the analytes in the tested range, 0.05–250 ng mL^−1^, which was wider than previously reported [3,9] All coefficients R^2^ were higher than 0.99 and are reported in Table 1. Lack-of-fit results also demonstrated the appropriateness of the linear model in the selected concentration range. The evaluation of the relative standard deviation of the slope was evaluated, a threshold of 5% was set, and it was accomplished for all of the analytes.

The absence of carryover was assessed given that, after the injection of the higher calibration sample (250 ng mL^−1^), the analysis of the blank did not show any signal above LOD at the analytes retention time.

The results obtained for analyte stability demonstrated that all analytes were stable in the tested conditions.

The method was highly selective since target analytes were not detected in all samples contaminated with a mixture containing other natural or synthetic opioids and some of their metabolites.

Intraday precision was evaluated by performing the analyses of QCs at three concentration levels in sextuplicate; good results were obtained with interday precision between 0% and 13%. Interday precision was evaluated by analyzing the same three QCs on three different working days; calculated RSD% was between 2% and 12% for all analytes. Results obtained for all of the analytes are reported in Table 2.

Accuracy values are shown in the same table and were between −13% and 19%.

Recoveries were between 43% for valerylfentanyl carboxy metabolite at low concentration level and 92% for despropionyl para-fluoro fentanyl, and recovery RSD% was <9% for all of the analytes. The matrix effect, evaluated on six different OF samples, and also on a pooled matrix, was <20%. Satisfying recoveries associated with good values of ME% reveal the applicability of the proposed procedure. Acceptable ME values were confirmed by post column infusion results in which no signal suppression zones were observed all along the chromatographic run.

Results are reported in Table 2.

### 2.4. Proficiency Samples Analysis

As a result of the participation of our laboratory to an interlaboratory proficiency test, three different OF samples, possibly containing fentanyls, were received.

To identify and quantify fentanyl and/or its derivatives and metabolites present in the samples, an aliquot was treated with the proposed method. One of the three samples was found to be positive for five different analytes, i.e., fentanyl, norfentanyl, acetylfentanyl, acetylnorfentanyl and carfentanyl.

Proficiency testing results were evaluated by means of the Z-score. Those scores were calculated as the difference between the result obtained in our laboratory and the population mean, divided by the standard deviation of the population. All Z-score values were lower than 1, as reported in Table 3, and considered satisfactory.

## 3. Materials and Methods

### 3.1. Chemicals and Reagents

Standard of acetylfentanyl (hydrochloride), acrylfentanyl (hydrochloride), alfentanil (hydrochloride), butyrylfentanyl, carfentanyl, (±)-cis-3-methylfentanyl (hydrochloride), (±)-cis-3-methylthiofentanyl (hydrochloride), fentanyl (hydrochloride), furanylfentanyl (hydrochloride), α-methylfentanyl (hydrochloride), α-methylthiofentanyl (hydrochloride), β-hydroxyfentanyl (hydrochloride), ortho-fluorofentanyl (hydrochloride), ocfentanyl, para-fluorofentanyl (hydrochloride), remifentanyl (hydrochloride), sufentanyl, 4-ANPP, norfentanyl, butyrylfentanyl carboxy metabolite, despropionylpara-fluorofentanyl, (±)-trans-3-methyl norfentanyl, methoxyacetyl norfentanyl (hydrochloride), valerylfentanyl carboxy metabolite, morphine, methadone, EDDP, 6-MAM, codeine, MT-45, fentanyl-d5 (hydrochloride) and nordentanyl-d5 were purchased from Cayman Chemical (Ann Arbor, MI, USA) in form of methanolic solution at the concentration of 1 mg mL^−1^. Working solutions were prepared at the concentration of 1 μg mL^−1^ and stored at −20 °C. Water, methanol, acetonitrile and 2-propanol were obtained from Carlo Erba (Milano, Italia) while sodium carbonate, sodium bicarbonate and ammonium formate were purchased from Sigma-Aldrich (Milwaukee, WI, USA).

### 3.2. Sample Preparation

Oral fluid (OF) was sampled by passive drool from 6 different volunteers, 3 males and 3 females, from 20 to 50 years old. All the volunteers declared to be in good health and to follow a balanced and heterogeneous diet; they were requested to abstain from eating, drinking and smoking at least for two hours before sampling collection. OF samples were collected and stored at −20 °C until they were treated and analyzed.

100 μL of OF were placed in an Eppendorf tube and 30 μL of MeCN/MeOH 40/60 containing the ISs at the final concentration of 25 ng mL^−1^ were then added. The obtained solution was vortex mixed for 1 min, then centrifuged at 17,500 g, 3 °C for 10 min. In total, 100 μL of the supernatant was diluted 1/1 with a carbonate buffer solution pH 11, then this solution was cleaned-up by means of a MEPS syringe (SGE Analytical Science—Grale HDS Sydney, Australia) equipped with a C18 barrel-in-needle (BIN). The cartridge was activated by flushing 3 times 250 μL of MeOH and then conditioned by flushing 3 times 250 μL of H_2_O/MeOH/MeCN 75/15/10. Afterwards, the sample was loaded and released five times to load the analytes in the cartridge. The latter was washed 3 times with water and finally elution of the analytes was performed by flushing 5 times 50 μL of MeOH 1% HCOOH. The eluate was diluted 1/1 with water and collected in vials, 3 μL were injected in the LC–HRMS/MS system for further analysis.

### 3.3. LC–MS Experimental Conditions

LC system was a Dionex Ultimate 3000 RSLC from Thermo Scientific (San Jose, CA, USA) equipped with autosampler, degasser and injection system with a 100 μL loop. Fentanyls and their metabolites were separated by means of an Ace Excel 2 column, (100 × 2.1 ID) packed with C18-PFP particles of 2.6 μm, from Advanced Chromatography Technologies Ltd. (Aberdeen, Scotland). The column was maintained at 35 °C with a Column Compartment TCC-3000SD (Thermo Scientific). Mobile phases were water 10 mM ammonium formate (phase A) and MeCN/MeOH (phase B) both acidified with 0.1% of HCOOH; flow rate was 0.5 mL min^−1^ and the elution was performed by means of the following gradient: phase B was initially held at 0% for 1 min, then phase B was increased to 25% in 2 min, subsequently to 35% in 2 min and held in this condition for 3 min; phase B was then increased to 50% in 1.5 min and finally to 100% in 1 min, and held at 100% for 1 min. The system returned to the initial conditions in 2 min. All analytes were eluted in 9.9 min.

The LC system was connected with a high-resolution mass spectrometer with Orbitrap technology, specifically a Q-Exactive Orbitrab from Thermo Scientific (Bremen, Germany) equipped with a heated electrospray ionization source (H-ESI) operating in positive ionization mode. Source parameters were set as follows: spray voltage 3.5 kV, capillary temperature 350 °C, H-ESI temperature 300 °C, nitrogen was used as sheat and auxiliary gasses and were, respectively, set at 55 and 25 units. The S-lens RF was set at 60. The MS instrument was calibrated every working day by means of a calibration mixture provided by Thermo Scientific. The selected acquisition mode was Parallel Reaction Monitoring (PRM), resolution was 35,000 FWHM, the Automatic Gain Control (AGC) target was 5 × 10^5^ and the related Maximum Injection Time (IT) was 80 ms. A window of 1 Da was selected for the isolation of the precursor ion. The Collision Energy (CE) was optimized for each analyte with Mass Traces scan of CE of the Tune Software (Thermo Scientific) by the direct injection in the mass spectrometer of a solution containing all the target analytes at the concentration of 15 ng mL^−1^. Spectra were obtained by the extraction of the accurate masses of the precursor ion and the two most intense fragment ions from the Total Ion Current (TIC). Quantitative analysis was performed by means of Xcalibur Quan Browser software (Thermo Scientific). Table 4 encloses a list of the analytes with their related retention time, the accurate mass of the precursor ion and the two most intense fragments.

### 3.4. Method Validation

Validation protocol was carried out in accordance with international guidelines. The following parameters were investigated: LODs and LOQs, linearity, accuracy and precision, selectivity, recovery and matrix effect, carryover and stability. Both calibration standards and quality control samples (QC) were prepared in a pool of drug-free OF, using samples obtained from five different subjects (blank OF).

QCs were prepared daily by spiking blank OF with a mixture containing all the analytes at three different concentrations. The investigated concentrations were 0.5, 25 and 250 ng mL^−1^.

For LODs and LOQs determination, 18 different blank OF samples were treated as described in §Sample preparation and fortified with different concentrations of the mixture containing the analytes, from 0.05 ng mL^−1^ to 10 ng mL^−1^ (*n* = 3). LODs were calculated as those concentrations that provided a signal-to-noise (S/N) equal or higher than 3/1. The same procedure was applied to LOQs’ determination, these values were defined as the lower concentration which provided an S/N higher than 10/1.

To evaluate linearity of the measuring interval, calibration curve was prepared by spiking blank OF with a mixture of the analytes at 7 different concentration levels (*n* = 3), i.e., 0.05, 0.1, 0.5, 2.5, 10, 50 and 250 ng mL^−1^. Internal standard ratio was used to evaluate the calibration samples analyzed in three different chromatographic runs and on three different working days. Thermo Xcalibur Quan Browser was used to perform the data regression using the weight of 1/x. To evaluate the linearity, lack-of-fit test was executed, as suggested by international guidelines.

Carryover was evaluated by performing the injection of three different blanks after the analysis of the highest concentration level of the calibration curve and verifying the absence of any signals.

Intraday and interday precision and accuracy were investigated on three different days, from the analysis of the QCs (*n* = 6). Precision was considerate acceptable when the RSD% was ±20%, while accuracy was calculated by means of bias and was required to be within 15%.

To evaluate recoveries and matrix effect, three sets of samples were prepared in triplicate at 0.5, 25 and 250 ng mL^−1^; the first set consisted of standard solutions in MeOH/H_2_O 50/50 (*v*/*v*) named S_(0.5)_, S_(25)_ and S_(250)_, the second set of samples was obtained by fortifying a pool of blank OF from five subjects after extraction (V_(0.5)_, V_(25)_ and V_(250)_) and the last set consisted of OF fortified before the extraction, these samples were identified as M_(0.5)_, M_(25)_ and M_(250)_. Recoveries (R%) were calculated as R% = (V_(X)_/M_(X)_) × 100. Matrix effect (ME%) was evaluated as follows: ME% = (V_(X)_/S_(X)_) × 100.

Matrix effect was also evaluated as recommended by the European Medicine Agency (EMEA) guidelines by post column infusion. To this aim, the analytes were separated in different groups of 5–6 analytes with different masses, solutions were prepared at 25 ng mL^−1^. The assessment of matrix effect was based on a constant infusion of the analytes solution (5 μL min^−1^) into the eluent from the analytical column when a blank OF was injected by a post column tee connection. In the absence of matrix interferences, the continuous post column infusion led to a constant signal in the detector, while elution of compounds that can enhance or suppress the analyte signal led to increased or decreased signal.

Stability was evaluated in the short-term at 4 °C (*t* = 1 h and *t* = 48 h), and in the long-term at −20 °C (*t* = 7 d, *t* = 14 d, *t* = 30 d). To this aim, a pooled OF blank sample was fortified at 25 ng mL^−1^ with the analytes and was split into several aliquots; five aliquots were immediately processed and analyzed (*t* = 0), while the other aliquots were analyzed at the different time points (*n* = 5). IS was added before processing in all the aliquots. Autosampler stability was evaluated by analyzing the extracts at *t* = 0, 2, 6 and 24 h.

Analyte stability was obtained at each time point by comparing the analyte/IS area ratio after storage to the one determined at *t* = 0. Analytes were considered stable in OF if their average respect to t0 were within ±20%.

Selectivity was investigated by fortifying a pool of OF samples with a mixture containing other illicit drugs, for this purpose a mixture containing morphine, methadone, EDDP, 6-MAM, codeine and MT-45 was added to the samples at a final concentration of 25 ng mL^−1^.

### 3.5. Proficiency Tests

To ascertain the applicability and verify the accuracy of the method, three samples were analyzed as described above. The samples were provided by the Istituto Superiore di Sanità (I.S.S.) and are part of a set of samples used in a national interlaboratory test (the Italian Valutazione Esterna di Qualità—V.E.Q.).

## 4. Conclusions

Since fentanyl and its analogues are increasingly used as drugs of abuse worldwide, it is crucial to provide fast and effective analytical methods to detect and quantify those compounds and their metabolites in biological matrices. OF is a useful matrix to asses psychoactive substances’ recent consumption, and is particularly useful for DUID cases. The presented method provides an easy sample preparation and good sensitivity. Sample pretreatment using MEPS offers numerous benefits, including the small amount of sample and solvent required, and a lower cost than conventional SPE. Furthermore, the method is also quite rapid since, from sample collection to data analysis, only 20 min per sample are required. The procedure can be easily applied in toxicology laboratories since processing several samples with this technique is quite simple, and, on the other hand, it is not essential to analyze the samples on the same day they are delivered to the laboratory, as demonstrated in the stability study.

The method was validated according to international guidelines with good results in terms of sensitivity, accuracy and precision, and allowed good recoveries for all the analyzed analytes to be obtained. In our opinion this method could represent a useful tool in forensic toxicology. Its applicability was demonstrated since it was successfully applied to the analysis of some samples provided from a proficiency test.

## Figures and Tables

**Table 1:** Validation data; LODs, LOQs and linearity.

Analyte	LOD (ng/mL)	LOQ (ng/mL)	Equation	R^2^
Methoxyacetyl Norfentanyl	0.10	0.50	y = −2.2 × 10^−4^ + 5.0 × 10^−2^x	0.993
Acetylnorfentanyl	0.20	1.0	y = −1.0 × 10^−4^ + 6.2 × 10^−2^x	0.996
Norfentanyl	0.20	0.50	y = −1.5 × 10^−4^ + 8.0 × 10^−2^x	0.995
+/−trans-3-methylnorfentanyl	0.10	0.20	y = −7.1 × 10^−4^ + 2.0 × 10^−1^x	0.996
Remifentanyl	0.10	0.20	y = −1.1 × 10^−4^ + 5.0 × 10^−2^x	0.994
Butyrylfentanil Carboxy Metabolite	0.50	1.0	y = −9.0 × 10^−5^ + 6.0 × 10^−2^x	0.994
Valerylfentanyl Carboxy Metabolite	0.20	0.50	y = −1.2 × 10^−4^ + 7.6 × 10^−2^x	0.994
Acetylfentanyl	0.10	0.50	y = −7.3 × 10^−4^ + 1.4 × 10^−1^x	0.995
Ocfentanyl	0.10	0.50	y = −5.8 × 10^−4^ + 1.4 × 10^−1^x	0.995
Beta-hydroxyfentanyl	0.10	0.50	y = −8.6 × 10^−6^ + 4.0 × 10^−2^x	0.996
Alfentanyl	0.10	0.50	y = −1.8 × 10^−4^ + 4.9 × 10^−2^x	0.994
Acrylfentanyl	0.05	0.20	y = −3.2 × 10^−4^ + 9.8 × 10^−2^x	0.997
alfa-methylthiofentanyl	0.10	0.20	y = −1.6 × 10^−2^ + 5.0 × 10^−2^x	0.997
Fentanyl	0.05	0.10	y = −4.7 × 10^−4^ + 9.3 × 10^−2^x	0.995
4-ANPP	0.05	0.50	y = −3.8 × 10^−5^ + 7.6 × 10^−2^x	0.997
+/−cis-3-methylthiofentanyl	0.05	0.50	y = −1.4 × 10^−2^ + 4.5 × 10^−2^x	0.998
Furanylfentanyl	0.05	0.10	y = −9.0 × 10^−4^ + 1.4 × 10^−1^x	0.994
+/−cis-3-methylfentanyl	0.10	0.50	y = −1.1 × 10^−3^ + 5.2 × 10^−2^x	0.997
para-Fluorofentanyl	0.20	1.0	y = −4.1 × 10^−1^ + 1.1 × 10^−1^x	0.994
ortho-Fluorofentanyl	0.20	1.0	y = −2.0 × 10^−1^ + 7.3 × 10^−2^x	0.994
Despropionyl para-Fluorofentanyl	0.10	0.50	y = −1.4 × 10^−3^ + 1.5 × 10^−1^x	0.995
alfa-methylfentanyl	0.10	0.50	y = −5.2 × 10^−4^ + 6.6 × 10^−2^x	0.997
Carfentanyl	0.20	0.50	y = −1.4 × 10^−3^ + 6.4 × 10^−2^x	0.996
Butyrylfentanyl	0.10	0.50	y = −4.5 × 10^−4^ + 1.2 × 10^−1^x	0.997
Sufentanyl	0.20	0.50	y = −3.5 × 10^−4^ + 2.9 × 10^−2^x	0.994

**Table 2:** Validation data; intraday precision, interday precision, accuracy, matrix effect and recoveries calculated at the selected three concentrations, 0.5, 25 and 250 ng mL^−1^.

Analyte	Concentration (ng/mL)	Intraday Precision (%)	Interday Precision (%)	Accuracy (%)	Matrix Effect (%)	Recovery (%)
Methoxyacetyl Norfentanyl	0.5	2	7	−3	119	57
	25	1	6	4	87	55
	250	3	7	−9	101	57
Acetyl Norfentanyl	0.5	4	12	5	99	46
	25	1	10	3	83	55
	250	3	11	−4	105	56
Norfentanyl	0.5	3	11	3	104	54
	25	0	5	−4	87	69
	250	3	12	−4	107	57
+/−trans-3-methyl Norfentanyl	0.5	1	7	−3	81	62
	25	2	2	−2	82	76
	250	3	10	−6	88	56
Remifentanyl	0.5	13	6	12	95	68
	25	11	2	6	94	75
	250	9	10	9	118	68
Butyrylfentanil Carboxy Metabolite	0.5	1	8	−9	111	59
	25	2	5	−4	87	63
	250	5	9	−11	106	58
Valerylfentanyl Carboxy Metabolite	0.5	1	8	13	116	43
	25	2	10	2	83	71
	250	4	11	−2	104	62
Acetylfentanyl	0.5	4	10	−9	115	54
	25	4	8	−10	107	84
	250	5	8	3	127	78
Ocfentanyl	0.5	0	7	17	96	73
	25	1	8	11	91	81
	250	3	11	6	115	77
Beta-hydroxyfentanyl	0.5	4	8	12	95	76
	25	3	7	2	89	80
	250	4	5	4	110	78
Alfentanyl	0.5	5	7	16	87	71
	25	1	7	4	85	87
	250	4	5	7	86	80
Acrylfentanyl	0.5	4	11	2	99	73
	25	2	9	−3	96	82
	250	3	4	3	121	75
alfa-methylthiofentanyl	0.5	4	8	2	98	82
	25	1	6	1	98	79
	250	3	7	−1	113	78
Fentanyl	0.5	5	9	−8	106	63
	25	3	3	−5	97	85
	250	3	6	−1	117	77
4-ANPP	0.5	9	6	−2	117	71
	25	4	3	−13	85	89
	250	3	7	−7	117	69
+/−cis-3-methylthiofentanyl	0.5	3	9	2	101	81
	25	2	9	−3	105	78
	250	2	8	−3	117	76
Furanylfentanyl	0.5	3	7	18	117	69
	25	1	2	9	101	82
	250	4	10	−1	118	79
+/−cis-3-methylfentanyl	0.5	7	7	−2	98	73
	25	1	5	4	85	79
	250	3	5	−4	99	79
para-Fluorofentanyl	0.5	9	10	11	95	81
	25	9	5	8	96	80
	250	2	2	18	115	77
ortho-Fluorofentanyl	0.5	9	7	11	99	79
	25	9	8	8	97	79
	250	7	2	18	117	78
Despropionyl para-Fluorofentanyl	0.5	2	7	−5	119	69
	25	1	3	4	80	92
	250	4	2	−8	114	71
alfa-methylfentanyl	0.5	7	6	9	107	75
	25	2	6	6	101	80
	250	3	8	−4	117	78
Carfentanyl	0.5	4	8	19	113	67
	25	1	9	4	95	87
	250	3	9	2	115	80
Butyrylfentanyl	0.5	7	11	2	120	61
	25	1	4	5	93	86
	250	4	2	−3	114	77
Sufentanyl	0.5	8	9	−3	99	74
	25	2	6	3	99	82
	250	2	5	−5	120	77

**Table 3:** Results obtained from proficiency test.

Analyte	Population Mean	Standard Deviation of the Population	Measurement Result of Our Laboratory	Z-Score
Fentanyl	38.10	12.99	42.45	0.33
Norfentanyl	81.65	14.03	76.08	−0.40
Acetylfentanyl	31.44	14.52	38.95	0.52
Acetylnorfentanyl	64.36	32.27	70.30	0.18
Carfentanyl	39.69	17.10	45.35	0.33

**Table 4:** LC–MS/HRMS parameters; formula, precursor ion, qualifier ion, quantifier ion and retention time for all the target analytes.

Analyte	Formula	Precursor Ion (m/z)	Qualifier Ion (m/z)	Quantifier Ion (m/z)	t_R_ (min)
Methoxyacetyl Norfentanyl	C_14_H_20_N_2_O_2_	249.1596	166.0862	84.0813	3.80
Acetylnorfentanyl	C_13_H_18_N_2_O	219.1490	136.0756	84.0812	3.88
Norfentanyl	C_14_H_20_N_2_O	233.1645	177.1384	84.0812	4.55
+/−trans-3-methylnorfentanyl	C_15_H_22_N_2_O	247.1803	150.0913	98.0968	5.31
Remifentanyl	C_20_H_28_N_2_O_5_	377.2065	113.0599	228.1229	5.62
Butyrylfentanil Carboxy Metabolite	C_23_H_28_N_2_O_3_	381.2621	363.2886	345.2418	5.98
Valerylfentanyl Carboxy Metabolite	C_24_H_30_N_2_O_3_	395.2329	246.1487	335.2114	6.16
Acetylfentanyl	C_21_H_26_N_2_O	323.2114	105.0700	188.1432	6.19
Ocfentanyl	C_22_H_27_FN_2_O_2_	371.2123	105.0700	188.1432	6.30
Beta-hydroxyfentanyl	C_22_H_28_N_2_O_2_	353.2220	335.2114	204.1382	6.35
Alfentanyl	C_21_H_32_N_6_O_3_	417.2607	268.1765	197.1283	7.15
Acrylfentanyl	C_22_H_26_N_2_O	335.1833	105.0700	188.1432	7.16
alfa-methylthiofentanyl	C_21_H_28_N_2_OS	357.1990	259.1802	208.1153	7.16
Fentanyl	C_22_H_28_N_2_O	337.2269	105.0700	188.1432	7.41
4-ANPP	C_19_H_24_N_2_	281.2008	150.0266	188.1433	7.64
+/−cis-3-methylthiofentanyl	C_21_H_28_N_2_OS	357.1990	259.1802	208.1153	7.74
Furanylfentanyl	C_24_H_26_N_2_O_2_	375.2061	105.0700	188.1431	8.03
+/−cis-3-methylfentanyl	C_23_H_30_N_2_O	351.2427	105.0700	202.1588	8.07
para-Fluorofentanyl	C_22_H_27_FN_2_O	355.2174	105.0700	188.1432	8.15
ortho-Fluorofentanyl	C_22_H_27_FN_2_O	355.2174	105.0700	188.1432	8.35
Despropionyl para-Fluorofentanyl	C_19_H_23_FN_2_	299.1926	105.0700	188.1432	8.80
alfa-methylfentanyl	C_23_H_30_N_2_O	351.2427	119.0857	202.1588	8.82
Carfentanyl	C_24_H_30_N_2_O_3_	393.2269	105.0700	188.1432	9.16
Butyrylfentanyl	C_23_H_30_N_2_O	351.2427	105.0700	188.1432	9.34
Sufentanyl	C_22_H_30_N_2_O_2_S	387.2093	238.1257	355.1833	9.90
